# High Prevalence of Hyper-Aerotolerant *Campylobacter jejuni* in Retail Poultry with Potential Implication in Human Infection

**DOI:** 10.3389/fmicb.2015.01263

**Published:** 2015-11-12

**Authors:** Euna Oh, Lynn McMullen, Byeonghwa Jeon

**Affiliations:** ^1^School of Public Health, University of AlbertaEdmonton, AB, Canada; ^2^Department of Agricultural, Food and Nutritional Science, University of AlbertaEdmonton, AB, Canada

**Keywords:** *Campylobacter jejuni*, aerotolerance, chicken isolates, MLST-genotyping, oxidative stress

## Abstract

*Campylobacter jejuni* is a leading cause of foodborne illnesses around the world. Since *C. jejuni* is microaerophilic and sensitive to oxygen, aerotolerance is important in the transmission of *C. jejuni* to humans via foods under aerobic conditions. In this study, 70 *C. jejuni* strains were isolated from retail raw chicken meats and were subject to multilocus sequence typing (MLST) analysis. In the aerotolerance testing by aerobic shaking at 200 rpm, 50 (71.4%) isolates survived after 12 h (i.e., aerotolerant), whereas 20 (28.6%) isolates did not (i.e., aerosensitive). Interestingly, further aerobic cultivation showed that 25 (35.7%) isolates still survived even after 24 h of vigorous aerobic shaking (i.e., hyper-aerotolerant). Compared to aerosensitive strains, the hyper-aerotolerant strains exhibited increased resistance to oxidative stress, both peroxide and superoxide. A mutation of *ahpC* in hyper-aerotolerant strains significantly impaired aerotolerance, indicating oxidative stress defense plays an important role in hyper-aerotolerance. The aerotolerant and hyper-aerotolerant strains were primarily classified into MLST clonal complexes (CCs)-21 and -45, which are known to be the major CCs implicated in human gastroenteritis. Compared to the aerosensitive strains, CC-21 was more dominant than CC-45 in aerotolerant and hyper-aerotolerant strains. The findings in this study revealed that hyper-aerotolerant *C. jejuni* is highly prevalent in raw chicken meats. The enhanced aerotolerance in *C. jejuni* would impact human infection by increasing possibilities of the foodborne transmission of *C. jejuni* under aerobic conditions.

## Introduction

*Campylobacter jejuni* is one of the leading bacterial causes of gastroenteritis (Altekruse et al., 1999), annually causing approximately 400–500 million infection cases worldwide (Ruiz-Palacios, 2007). *C. jejuni* is a commensal bacterium in a wide range of animals and is zoonotically transmitted to humans mainly by the consumption of contaminated animal products (Nielsen et al., 2006; Wilson et al., 2008). Particularly, high colonization levels of *C. jejuni* in the poultry intestines often result in the contamination of poultry products during processing, and contaminated poultry is the major source of transferring *C. jejuni* to humans (Silva et al., 2011). In Canada, 62% of retail raw chicken legs are contaminated with *Campylobacter* (Bohaychuk et al., 2006). In UK and US, similarly, *Campylobacter* is found in 76 and 57.3% of retail oultry products, respectively (Cui et al., 2005; Little et al., 2008). The transmission of *Campylobacter* is also caused by contamination through food handlers and cross-contamination involving contaminated kitchen equipment (Medeiros et al., 2008; Kennedy et al., 2011). *Campylobacter* is also isolated from various environmental sources, such as wildlife, sewage, and manure, which may work as vehicles to disseminate *Campylobacter* in food production systems and to humans (Whiley et al., 2013). In both foodborne and environmental routes of *C. jejuni* transmission to humans, *C. jejuni* should overcome harsh environmental conditions, particularly high oxygen tensions in atmosphere, which may directly impact the viability of *C. jejuni*.

As a thermotolerant and microaerophilic bacterium, *C. jejuni* grows optimally at 42°C under low oxygen environments (Young et al., 2007). Although *C. jejuni* is sensitive to high oxygen tensions in atmosphere, it exhibits a certain level of aerotolerance to survive under oxygen-rich conditions (Kaakoush et al., 2007; Luber and Bartelt, 2007). Aerotolerance is closely related to oxidative stress defense, since aerobic exposure results in the accumulation of toxic reactive oxygen species (ROS) that may give damage to proteins and lipids in *C. jejuni* (Oh et al., 2015). A few genes of oxidative stress defense have been shown to affect aerotolerance in *C. jejuni*. For instance, a mutation of *fdxA* encoding the ferredoxin FdxA significantly reduces aerotolerance in *C. jejuni* (van Vliet et al., 2001). A double mutation of bacterioferritin comigratory protein (i.e., Bcp) and thiol peroxidase (i.e., Tpx) impairs the aerotolerance of *C. jejuni* (Atack et al., 2008). Recently, we reported that alkyl hydroperoxide reductase (AhpC) plays a more important role in the aerotolerance of *C. jejuni* than other key ROS-detoxification enzymes, such as catalase (KatA) and superoxide dismutase (SodB; Oh et al., 2015).

Presumably due to our traditional notion that *C. jejuni* is sensitive to oxygen, aerotolerance has not been investigated extensively in *C. jejuni* isolates from poultry. To examine the level of aerotolerance in *C. jejuni*, in the present study, we isolated 70 *C. jejuni* strains from retail chicken meats in Edmonton, Alberta, and analyzed their aerotolerance, revealing that aerotolerant and hyper-aerotolerant *C. jejuni* strains are highly prevalent in chicken. Moreover, most hyper-aerotolerant *C. jejuni* isolates belong to the clonal complexes (CCs) of multilocus sequence typing (MLST) that are frequently implicated in human infection and outbreaks.

## Materials and Methods

### Isolation of *C. jejuni* from Retail Chicken Meats

Chicken meats of different brands and products were purchased from seven different retail stores in Edmonton, Canada. *C. jejuni* was isolated as described by Chon et al. (2012) with some modifications. Briefly, the chicken meats were submerged in buffered peptone water (Oxoid, UK) at 37°C overnight and then inoculated in Bolton *Campylobacter* selective broth (Oxoid) at 42°C for 24 h under microaerobic conditions (5% O_2_, 10% CO_2_, 85% N_2_). Aliquots (100 μl) were serially diluted and spread on Mueller-Hinton (MH) agar plates supplemented with Preston *Campylobacter* selective supplements (Oxoid). Cultures were incubated at 42°C for 48 h under microaerobic conditions. *C. jejuni* colonies were confirmed by multiplex PCR as described previously (Wang et al., 2002). The primer sequences were described in **Table 1**. All *C. jejuni* strains were grown on MH media at 42°C under microaerobic conditions. Occasionally, culture media were supplemented with kanamycin (50 μg/ml).

**Table 1:** Primers used in this study.

Target gene	Primer	Sequence (5′–3′)	Reference
*C. jejuni hipO*	Jejuni_F Jejuni_R	ACTTCTTTATTGCTTGCTGC GCCACAACAAGTAAAGAAGC	Wanget al., 2002
*C. coli glyA*	Coli_F Coli_R	GTAAAACCAAAGCTTATCGTG TCCAGCAATGTGTGCAATG	Wang et al., 2002
*C. lari glyA*	Lari_F Lari_R	TAGAGAGATAGCAAAAGAGA TACACATAATAATCCCACCC	Wang et al., 2002
*aspA*	aspA_F aspA_R	AGTACTAATGATGCTTATCC ATTTCATCAATTTGTTCTTTGC	Dingle et al., 2001
*glnA*	glnA_F glnA_R	TAGGAACTTGGCATCATATTACC TTGGACGAGCTTCTACTGGC	Dingleet al., 2001
*gltA*	gltA_F gltA_R	GGGCTTGACTTCTACAGCTACTTG CCAAATAAAGTTGTCTTGGACGG	Dingle et al., 2001
*glyA*	gly_F gly_R	GAGTTAGAGCGTCAATGTGAAGG AAACCTCTGGCAGTAAGGGC	Dingleet al., 2001
*tkt*	tkt_F tkt_R	GCAAACTCAGGACACCCAGG AAAGCATTGTTAATGGCTGC	Dingle et al., 2001
*pgm*	pgm_F pgm_R	TACTAATAATATCTTAGTAGG CACAACATTTTTCATTTCTTTTTC	Dingle et al., 2001
*uncA*	uncA_F uncA_R	ATGGACTTAAGAATATTATGGC ATAAATTCCATCTTCAAATTCC	Dingle et al., 2001
*ahpC*	mahpC-F mahpC-R	CATGATAGTTACTAAAAAAGCTTTAG GTTAAAGTTTAGCTTCGTTTTTGCC	Oh and Jeon, 2014

### MLST Analysis

MLST analysis of *C. jejuni* isolates was performed based on the method outlined in pubMLST (pubmlst.org) and a previous report (Dingle et al., 2001) by using seven housekeeping genes, including *aspA*, *glnA*, *gltA*, *glyA*, *pgm*, *tkt*, and *uncA* (**Table 1**). Overnight culture of *C. jejuni* on MH agar plates was harvested in 1 ml of PBS, and then 10 μl of *C. jejuni* suspension was mixed with 90 μl of PBS and boiled for 10 min. After centrifugation, pellets were removed, and supernatant was used as template. PCR was carried out with ExTaq polymerase (Takara, Japan). PCR amplicons were commercially sequenced by Macrogen (Seoul, Korea), and the sequences were analyzed in the *Campylobacter* PubMLST database (http://pubmlst.org/campylobacter/).

### Aerotolerance Test

Aerotolerance test was carried out according to our previous report (Oh et al., 2015). Briefly, *C. jejuni* strains were grown on MH agar at 42°C for 18 h under microaerobic conditions. *C. jejuni* strains were resuspended in fresh MH broth and diluted to an OD_600_ of 0.07, and then bacterial suspensions were incubated at 42°C with shaking at 200 rpm under aerobic conditions. Samples were taken after 0, 12, and 24 h for serial dilution and CFU counting.

### Susceptibility to Oxidative Stress

*Campylobacter jejuni* strains were inoculated in MH broth at 42°C for 8 h with agitation under microaerobic conditions, and then bacterial cultures were exposed for 1 h to oxidants, including 100 μM of cumene hydroperoxide (CHP), 1 mM of hydrogen peroxide (H_2_O_2_) and 100 μM menadione (MND; a superoxide generator). The viability was determined by serial dilution and CFU counting.

### Construction of *ahpC* Mutants

A suicide plasmid for an *ahpC* mutation in *C. jejuni* was described in our previous study (Oh and Jeon, 2014). The *ahpC* suicide vector was introduced to *C. jejuni* strains by electroporation, and *ahpC* mutants were selected by growing on MH agar supplemented with kanamycin (50 μg/ml). The *ahpC* mutation was also confirmed by PCR with mahpC-F and mahpC-R primers (**Table 1**).

### Statistical Analysis

Statistical analysis was carried out using GraphPad Prism 6 (GraphPad Software, inc., USA). Statistical significance of differences between the groups was compared by using two-way analysis of variance (ANOVA). The frequency distribution was determined as described elsewhere (Callicott et al., 2008) by performing the Mann–Whitney test followed by one-way ANOVA. n.s.: *P* > 0.5, ^∗^*P* < 0.05, ^∗∗^*P* < 0.01, ^∗∗∗^*P* < 0.001, ^∗∗∗∗^*P* < 0.0001.

## Results

### MLST Analysis of *C. jejuni* Isolates from Retail Chicken Meats

We isolated 70 *C. jejuni* strains from 19 raw chicken meats from seven different retail stores in Edmonton, Alberta. The majority (77.1%) of *C. jejuni* strains was isolated from whole chicken samples, and 18.6% from drumsticks, and 4.3% from thighs (**Table 2**). The MLST results showed that the 70 strains were distributed in six different CCs. The major CCs were 21 and 45 that constituted 42.86 and 21.43% of *C. jejuni* isolates from chickens, respectively (**Table 2** and Supplementary Figure S1).

**Table 2:** Clonal complexes of *C. jejuni* isolates from raw chicken meats.

Clonal complex	ST no.	Isolate number	Sources
21	13	66	Whole chicken
	21	23	Whole chicken
		24	Whole chicken
		32	Whole chicken
		33	Whole chicken
		34	Whole chicken
		36	Whole chicken
		37	Whole chicken
	43	29	Thigh
		44	Whole chicken
	50	25	Whole chicken
		30	Whole chicken
		31	Whole chicken
		35	Whole chicken
		38	Whole chicken
	806	62	Whole chicken
		63	Whole chicken
		64	Whole chicken
		65	Whole chicken
	1086	8	Whole chicken
		43	Whole chicken
	2375	13	Whole chicken
	2377	68	Drumstick
	3794	10	Whole chicken
	4663	15	Drumstick
		16	Drumstick
	4681	11	Whole chicken
		12	Whole chicken
	4911	67	Whole chicken
	6261	7	Whole chicken
UA^a^	1698	48	Drumstick
		49	Drumstick
		50	Drumstick
		69	Drumstick
		70	Drumstick
	934	54	Whole chicken
	158	28	Drumstick
	1352	55	Thigh
45	45	52	Whole chicken
		53	Whole chicken
		56	Whole chicken
		57	Whole chicken
		58	Whole chicken
	137	2	Whole chicken
		4	Whole chicken
	659	1	Whole chicken
	998	27	Drumstick
	1818	21	Whole chicken
		22	Whole chicken
	6193	26	Drumstick
		59	Whole chicken
		60	Whole chicken
		61	Whole chicken
362	587	3	Whole chicken
		14	Whole chicken
		39	Whole chicken
		40	Whole chicken
		41	Whole chicken
		42	Whole chicken
353	452	6	Whole chicken
		9	Whole chicken
	3484	45	Whole chicken
354	2359	5	Whole chicken
48	142	51	Thigh
NT^b^		17	Whole chicken
		18	Whole chicken
		19	Whole chicken
		20	Whole chicken
		46	Drumstick
		47	Drumstick

*^a^UA: isolates that were unassigned to any CC defined.*

*^b^NT: not typable.*

### Differential Aerotolerance Levels in *C. jejuni* Chicken Isolates

**FIGURE 1:**
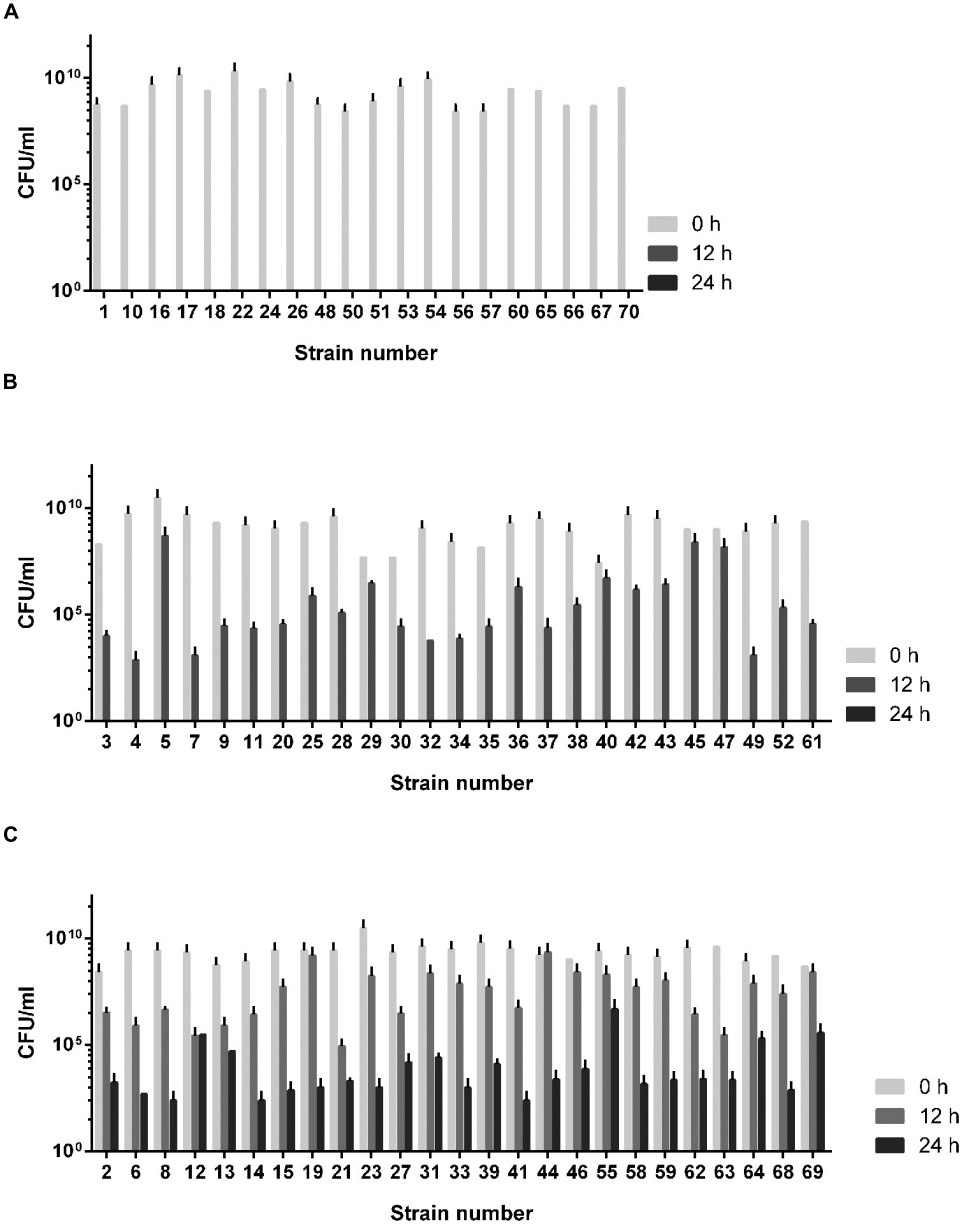
**The aerotolerance levels of *Campylobacter jejuni* isolates from raw chicken meats. (A)** Aerosensitive strains lost the viability prior to 12 h of aerobic shaking. **(B)** Aerotolerant strains maintained viability 12∼24 h of aerobic shaking. **(C)** Hyper-aerotolerant strains remained viable after 24 h of aerobic ∼shaking at 200 rpm. The results show the means and standard deviations of three different experiments. The aerotolerance tests were repeated at least three times, and similar results were observed in all the experiments.

**FIGURE 2:**
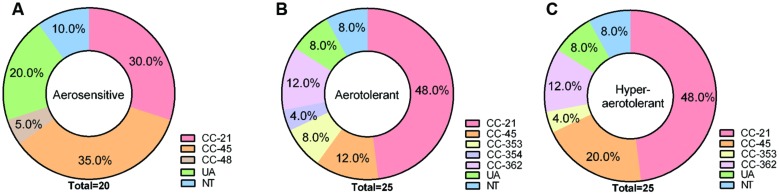
**Multilocus sequence typing (MLST) clonal complexes of *C. jejuni* isolates in three different aerotolerance groups; (A) aerosensitive, (B) aerotolerant, (C) hyper-aerotolerant groups**.

The aerotolerance of the 70 *C. jejuni* isolates from chickens was investigated by growing them aerobically with vigorous shaking at 200 rpm. Depending on the levels of aerotolerance, the 70 isolates were clustered into three groups: (i) the aerosensitive group that lost viability before 12 h (**Figure 1A**), (ii) the aerotolerant group in which *C. jejuni* strains maintained viability for 12∼24 h (**Figure 1B**), and (iii) the hyper-aerotolerant group where *C. jejuni* strains remained viable even after 24 h of aerobic shaking (**Figure 1C**). Whereas a total of 20 strains were aerosensitive, most isolates were aerotolerant or hyper-aerotolerant (**Figure 1**). Interestingly, 25 *C. jejuni* strains were hyper-aerotolerant and maintained viability even after 24 h of vigorous shaking (at 200 rpm) under aerobic conditions (**Figure 2C**). The major CCs were CC-45 (35%) and CC-21 (30%) in the aerosensitive group (**Figure 2A**). However, CC-21 was predominant in both aerotolerant and hyper-aerotolerant groups; 48% in each (**Figures 2B,C**). No *C. jejuni* isolates in CC-353 and CC-362 were aerosensitive, but they constituted about 16% of hyper-aerotolerant *C. jejuni* isolates (**Figures 2A,C**). These data show high prevalence of hyper-aerotolerant *C. jejuni* in retail chicken meats, which clusters mainly in a few major CCs.

### Oxidative Stress Resistance in *C. jejuni* Isolates

**FIGURE 3:**
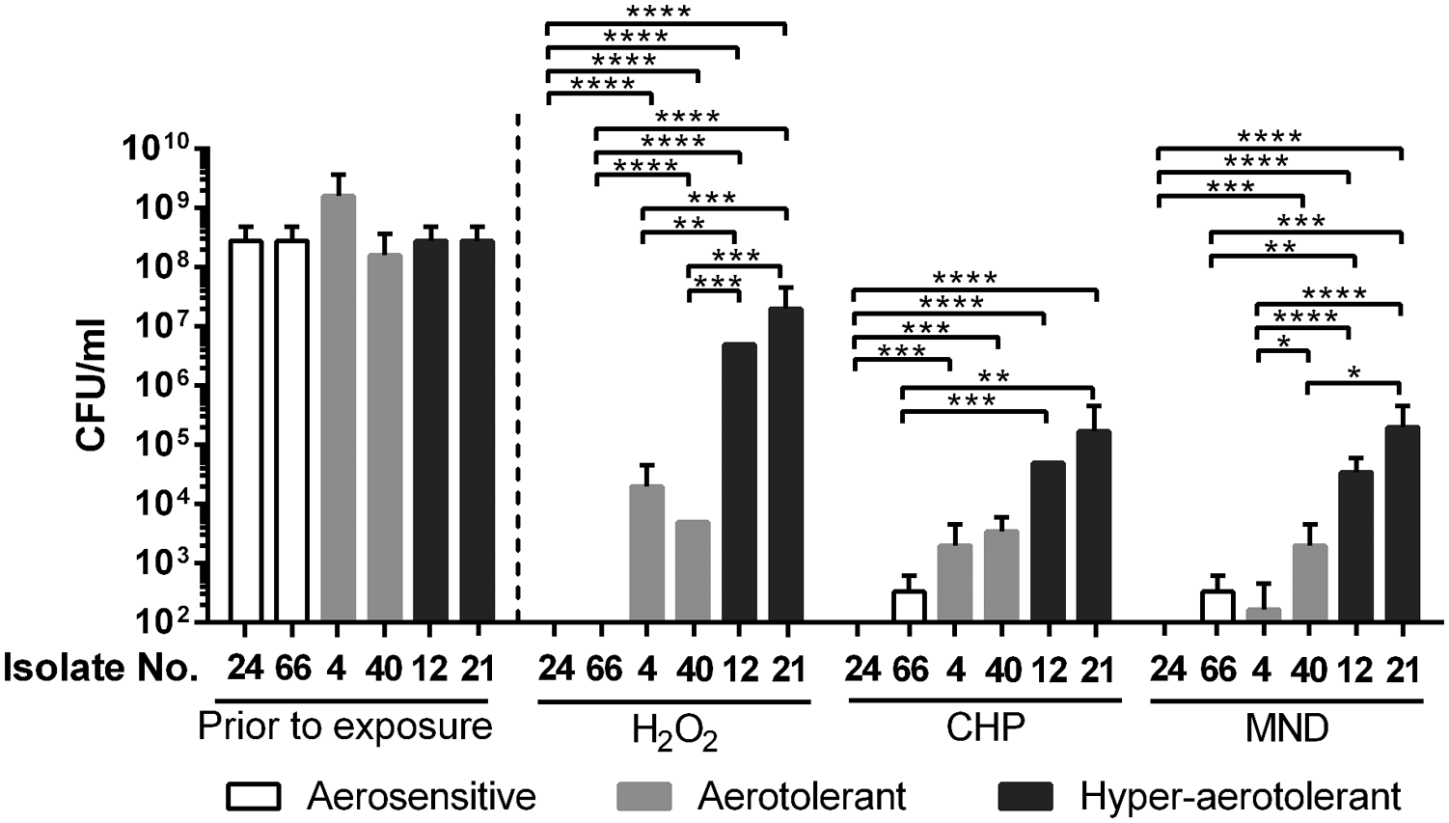
**Oxidative stress resistance of *C. jejuni* isolates from different aerotolerance groups.** The results show the CFU levels after 1 h exposure to oxidants, including 1 mM hydrogen peroxide (H_2_O_2_), 100 μM cumene hydroperoxide (CHP), and 100 μM menadione (MND). Statistical significance was analyzed with two-way ANOVA using GraphPad Prism 6 (GraphPad Software, inc.). ^∗^*P* < 0.05, ^∗∗^*P* < 0.01, ^∗∗∗^*P* < 0.001, ^∗∗∗∗^*P* < 0.0001.

Since oxidative stress defense plays an important role in the aerotolerance of *C. jejuni* (Oh et al., 2015), oxidative stress resistance was compared between the different aerotolerance groups. Two strains from each aerotolerance group were randomly chosen and exposed to different kinds of oxidants for 1 h for viability testing. The strains from the aerosensitive group easily lost viability by exposure to H_2_O_2_, CHP, and menadione (**Figure 3**). However, two strains from the hyper-aerotolerance group demonstrated significantly enhanced resistance to oxidative stress, both peroxide and superoxide (**Figure 3**). The strains from the aerotolerant group were more resistant to oxidants than the strains from the aerosensitive group but more susceptible than those from the hyper-aerotolerant group (**Figure 3**). The results clearly indicate that hyper-aerotolerant strains are more resistant to oxidative stress than aerosensitive strains.

### Role of *ahpC* in the Aerotolerance of Hyper-aerotolerant *C. jejuni* Isolates

**FIGURE 4:**
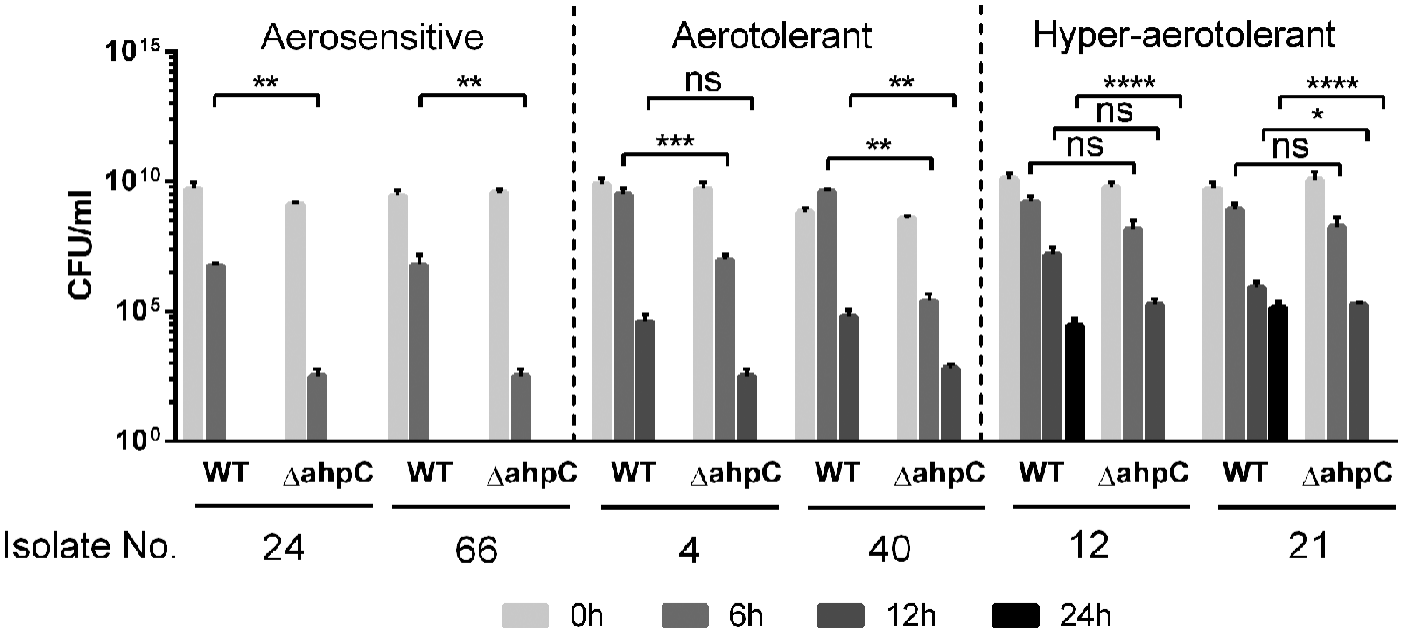
**Growth defects in the *ahpC* mutants under aerobic conditions.** The results show the CFU levels of triplicate samples after 6, 12, and 24 h culture under aerobic conditions with shaking at 200 rpm. The strains were selected from the three different aerotolerance groups. The experiment was repeated at least three times, and similar ∼results were ∼observed in all experiments. Statistical significance was determined with two-way ANOVA using GraphPad Prism 6 (GraphPad Software, inc.). n.s.: *P* > 0.5, ^∗^*P* < 0.05, ^∗∗^*P* < 0.01, ^∗∗∗^*P* < 0.001, ^∗∗∗∗^*P* < 0.0001.

Our previous study showed that *ahpC* plays a key role in *C. jejuni* survival under aerobic conditions, compared to other ROS-detoxification genes, such as *katA* and *sodB* (Oh et al., 2015); thus, we decided to investigate how *ahpC* contributes to hyper-aerotolerance in *C. jejuni*. We constructed *ahpC* knockout mutants of the six *C. jejuni* strains from the three different aerotolerance groups. The *ahpC* mutation in the strains from the aerosensitive group resulted in approximately 3 log CFU reduction compared to wild type after 6 h of aerobic culture (**Figure 4**). Interestingly, *ahpC* mutants of the hyper-aerotolerant strains did not survive after 24 h, indicating the *ahpC* mutation reduced the aerotolerance level from hyper-aerotolerance to aerotolerance. These findings clearly showed that oxidative stress defense, particularly *ahpC*, is critical for the hyper-aerotolerance in *C. jejuni*.

## Discussion

The poultry intestines provide optimal growth conditions for *C. jejuni*, such as high body temperature (42°C), high nutrients, and low oxygen levels. However, *C. jejuni* is exposed to oxygen-rich conditions during food processing and preservation. Thus, oxidative stress is an unavoidable stress that *C. jejuni* should overcome to survive during its foodborne transmission to humans (Kim et al., 2015). Despite our common perception that *C. jejuni* is sensitive to oxygen, in this study, we revealed that hyper-aerotolerant *C. jejuni* is highly prevalent in raw chicken meats. *Arcobacter* sp. are similar to *Campylobacter* and previously described as an aerotolerant *Camplyobacter*-like organism. *Arcobacter* is associated with animals and humans, and is frequently isolated in poultry (Vandamme et al., 1992; Snelling et al., 2006). Therefore, specific identification methods are needed to differentiate between *Campylobacter* and *Arcobacter* (Call et al., 2003), and multiplex PCR is often used for this purpose (Mandrell and Wachtelt, 1999). The *hipO* gene encoding hipuricase is present only in *C. jejuni* but not in any other *Campylobacter* sp. and *Arcobacter* sp. (Wang et al., 2002; Jensen et al., 2005). Whereas *C. jejuni* preferably grows at 42°C, *Arcobacter* grows optimally 24∼30°C (Phillips, 2001). In this study, *C. jejuni* was isolated at 42°C, at which *Arcobacter* cannot grow (Bhunia, 2008). In addition, the isolates were confirmed by PCR with primers for the *hipO* gene and classified by the MLST scheme for *C. jejuni*.

Most *C. jejuni* isolates from raw chicken meats belonged to CC-21 (42%) and CC-45 (21%; **Table 2** and Supplementary Figure S1). This is consistent with previous reports from Canada and other countries. The ST-21 and ST-45 complexes are most dominant in a variety of sources around the world, accounting for 39 and 14% *C. jejuni* isolates deposited in pubMLST, where 76% of isolates in the CC ST-21 and 60% in the CC ST-45 are of human origin, such as stool and blood (Colles and Maiden, 2012). In the UK, CCs ST-21 and ST-45 are commonly detected in veterinary (i.e., cow, pet, sheep, and poultry) and human sources (Manning et al., 2003). CCs 21, 45, 353, and 354 are also highly prevalent in sources from humans and foods in Eastern China (Zhang et al., 2015). According to the results of an MLST analysis of 122 *C. jejuni* isolates from Danish patients with symptoms of gaostroenteritis, reactive arithritis, and Guillain–Barré syndrome, CCs ST-21, -45, and -22 are highly prevalent, accounting for 64% of all *C. jejuni* isolates from humans. Whereas ST-22 is significantly linked to Guillain–Barré syndrome, ST-21 and -45 are primarily associated with gastroenteritis (Nielsen et al., 2010). An extensive MLST analysis of 289 *C. jejuni* isolates in Canada showed that CCs 21, 45, and 353 constitute the major *C. jejuni* population (CC-21: 26%, CC-45: 18%, and CC-353: 11%) in a variety of sources, including humans, chickens, raw milk, and environmental water. Interestingly, more than 50% of isolates that were classified in CC-21 were from humans (Lévesque et al., 2008), indicating high frequencies of human infection by *C. jejuni* strains in CC-21. In the US, an MLST analysis of 47 *C. jejuni* isolates from 12 outbreaks exhibited that CC ST-21 is commonly involved in human epidemics (Sails et al., 2003). Whereas CC-21 and CC-45 were distributed at similar levels in the aerosensitive *C. jejuni* isolates (CC-21: 30%, CC-45: 35%; **Figure 2A**), CC-21 is predominant in both aerotolerant (48%) and hyper-aerotolerant (48%) isolates (**Figures 2B,C**), and the frequencies of CC-21 distribution in aerotolerant and hyper-aerotolerant groups were statistically significant (Supplementary Figure S2).

Hyper-aerotolerant *C. jejuni* strains were more resistant to peroxide (i.e., H_2_O_2_ and CHP) and superoxide (i.e., menadione) stresses than aerosensitive strains (**Figure 3**), suggesting that increased resistance to oxidative stress would contribute to hyper-aerotolerance in *C. jejuni*. Our previous study showed that *ahpC* is the most important ROS-detoxification gene in the aerotolerance of *C. jejuni* (Oh et al., 2015). In this study, we demonstrated that *ahpC* is also a key player in *C. jejuni*’s hyper-aerotolerance (**Figure 4**). Enhanced resistance to oxidative stress may result in hyper-aerotolerance; this enables hyper-aerotolerant *C. jejuni* to survive easily under aerobic conditions during food processing, increasing risks of foodborne transmission to humans. Interestingly, it has been reported that *C. jejuni* strains in CC-21 from poultry and human clinical samples frequently exhibit hyper-invasiveness than strains in other CCs (Fearnley et al., 2008). *C. jejuni* strains in CC-21 usually (85.7%) have the sialylated lipooligosaccharide (LOS) class C and are highly invasive than isolates from other CCs (Habib et al., 2009). Taken our findings and these previous reports together, *C. jejuni* strains in CC-21 are often hyper-aerotolerant and likely to be invasive. Presumably, this would be why CC-21 is frequently involved in human infection and outbreaks (Sails et al., 2003; Nielsen et al., 2010).

To be the best of our knowledge, this is the first report about the prevalence of hyper-aerotolerant *C. jejuni* in chicken meats. Importantly, hyper-aerotolerant *C. jejuni* isolates are distributed mostly in the CCs that are often implicated in human infection, suggesting potential impact of hyper-aerotolerance on food safety and public health. Although we showed oxidative stress defense contributes to hyper-aerotolerance in *C. jejuni* in this study, there seems to be other unknown factors associated with hyper-aerotolerance since *ahpC* reduced aerotolerance, but not completely eliminated it (**Figure 4**). Based on the differential clustering of the LOS class in CC-21 (Habib et al., 2009), possibly some other genetic variations might also be involved in hyper-aerotolerance. Therefore, an extensive future study, such as whole genome sequencing, will be needed to further characterize hyper-aerotolerant *C. jejuni* and to reveal its implication in human infection.

## Author Contributions

Design of the project: EO, LM, and BJ; Performance of the experiments: EO; Data analysis: EO, LM, and BJ; Writing of the manuscript: EO, LM, and BJ.

## Conflict of Interest Statement

The authors declare that the research was conducted in the absence of any commercial or financial relationships that could be construed as a potential conflict of interest.
