# Supplementary material for: High Prevalence of Hyper-Aerotolerant Campylobacter jejuni in Retail Poultry with Potential Implication in Human Infection
Source: Front Microbiol. 2015 Nov 12;6:1263. doi: 10.3389/fmicb.2015.01263 (PMC4641907; doi:10.3389/fmicb.2015.01263)
Supplement: Supplementary file 1 [file Presentation_1.PDF]

Fig. S1

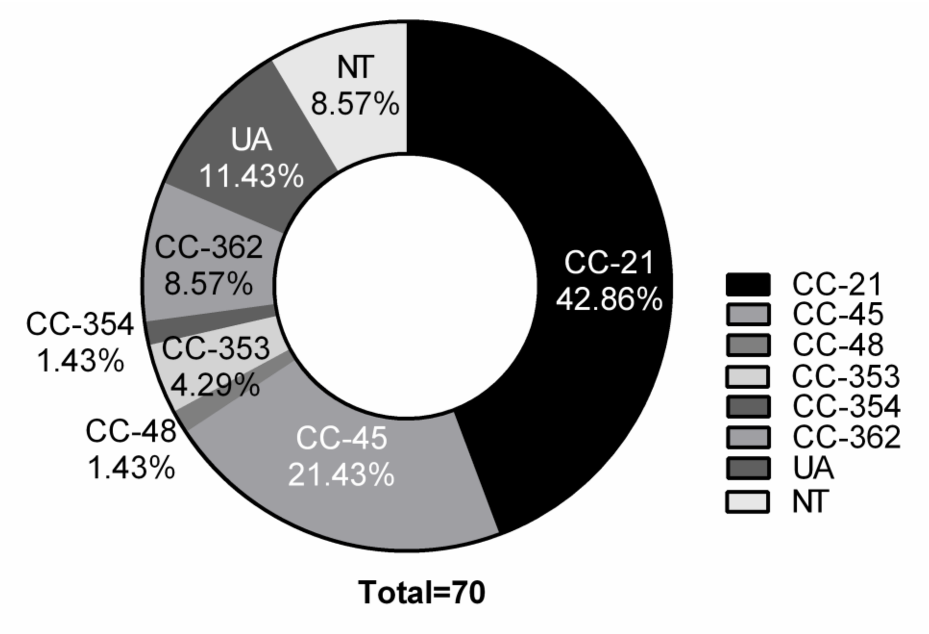

Fig. S1. Clonal complexes (CCs) of *C. jejuni* isolates from raw chicken meats. UA indicates the isolates that were not assigned to any CCs. NT is non-typable strains.

Fig. S2

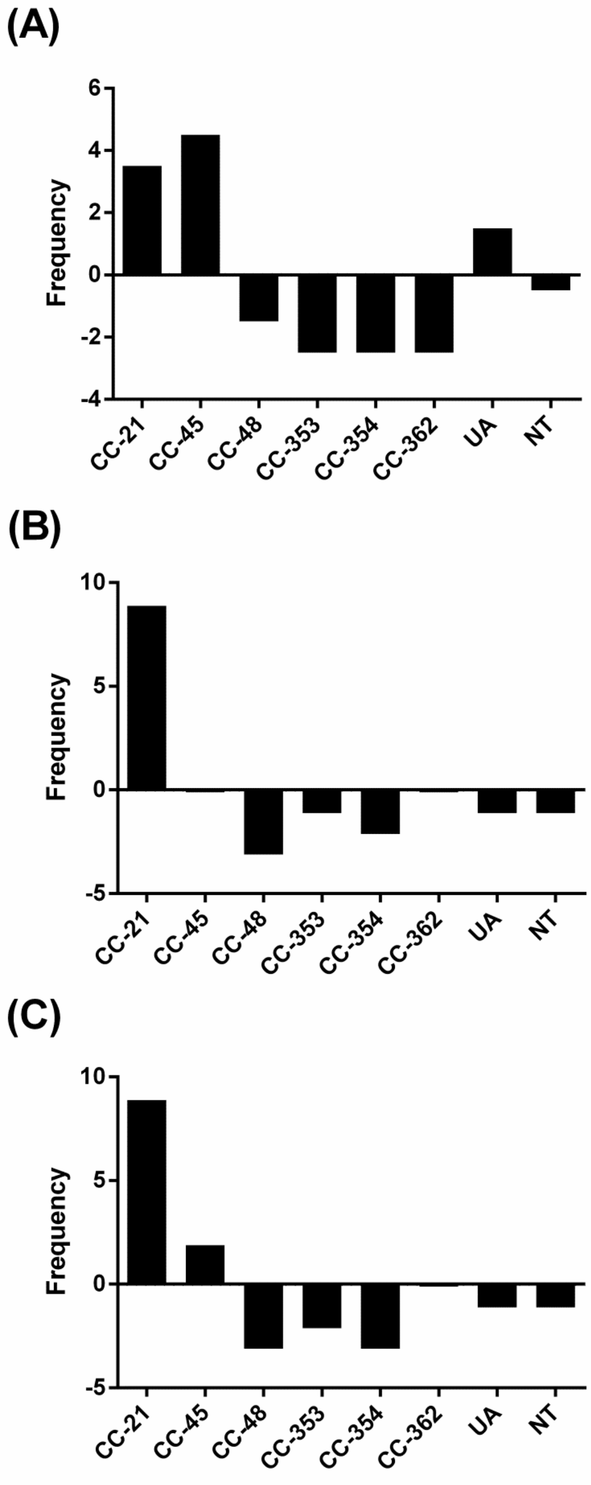

Fig. S2. The frequency distribution of *C. jejuni* isolates in three different aerotolerant groups. The frequency distribution was analyzed by the Mann-Whitney test, and then the results were analyzed by one-way ANOVA on each group: aerosensitive (A), aerotolerant (B), and hyper-aerotolerant (C) groups. The statistical analysis was performed using GraphPad Prism 6 (GraphPad Software Inc., USA)
